# The Role of Somatic Cell Synchronization in Nuclear Transfer and Induced Pluripotent Stem Cells for Wild Felids

**DOI:** 10.1002/zoo.21896

**Published:** 2025-03-07

**Authors:** João V.S. Viana, Alexsandra F. Pereira

**Affiliations:** ^1^ Laboratory of Animal Biotechnology Federal Rural University of Semi‐Arid Mossoró Brazil

**Keywords:** cloning, conservation strategies, fibroblast, nuclear reprogramming, wildlife

## Abstract

Human interference reduces wild felid populations. Somatic cell nuclear transfer and the use of induced pluripotent stem cells are potential conservation strategies. To improve the efficiency of these strategies, it is essential to establish adequate protocols for the synchronization of cells in the G_0_/G_1_ phase of the cell cycle. Cell cycle synchronization can arrest cell cycle progression by inhibiting factors involved in cell duplication. However, this step varies among wild felids and has not been successful in some species. In addition, the effect of this step on cell applications remains unclear. Therefore, this review highlights the primary differences among wild felids that can cause this variability, the most promising results, and the methods used. Finally, the importance of cell cycle synchronization in biotechnologies involving the nuclear reprogramming of somatic cells in wild felid conservation is highlighted.

## Graphical abstract

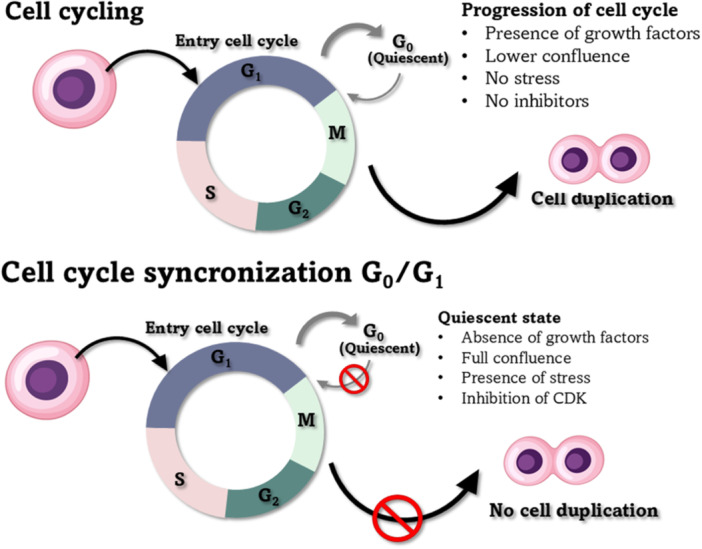

## 1 Introduction

Human interference disrupts the natural balance and decreases mammalian populations. This is most evident in the Felidae family, with 47% of species in this family categorized as in threat of extinction (Fernández‐Sepúlveda and Martín 2022). Population reductions of these species have been accompanied by ecosystem imbalances, especially in nutrient distribution and genetic diversity (Karandikar et al. 2022). Therefore, advances in conservation methods are essential.

*In situ* and *ex situ* conservation strategies have been developed and are correlated with increased biodiversity (Luczinski et al. 2023). Focusing on *ex situ* conservation, several countries have studied new possibilities for implementing associative systems between zoos and storing biological materials for use in assisted reproductive technologies (ARTs; Kochan et al. 2019; Swanson 2023). Embryos, gametes, and gonadal tissues are the first choices for animal conservation, and advances in cloning via somatic cell nuclear transfer (SCNT; Veraguas et al. 2017) and the production of induced pluripotent stem cells (iPSCs; Verma et al. 2013) have promoted the establishment of fibroblast cryobanks for wild felids.

In general, using fibroblasts in reprogramming techniques requires synchronization of the cell cycle in G_0_/G_1_. The cell cycle is defined by a series of events regulated by a group of cyclin‐dependent protein kinases (CDKs) that promote the correct progression to the G_1_, S, G_2_, and M phases. The quiescent state of a cell is promoted during cell cycle synchronization (Wang 2021). Cell cycle arrest correlated with increased homogeneity and CDK regulation, resulting in improved vector infection for iPSC generation (Zhao et al. 2016). Similarly, cell cycle arrest is necessary for SCNT to oocytes in metaphase II and somatic cells, allowing for the correct replication of genetic material (Thongphakdee et al. 2020).

Several cell cycle synchronization methodologies have been applied, based on chemical agents (Hashem et al. 2007), contact inhibition (CI), or serum starvation (SS) (Młodawska et al. 2022). Different methods promote the inhibition of cell proliferation via the suppression of CDK or other mechanisms. Promising results have been observed in wild felids when using chemical agents with a G_0_/G_1_ cycle synchronization efficiency of 80% and CI and SS values of approximately 85%–95% in some species (Wittayarat et al. 2013; Yelisetti et al. 2016). However, the results have been highly variable, and for some species, all methodologies have been ineffective, with percentages less than 75% (Hashem et al. 2007).

Although protocols for cell cycle synchronization in G_0_/G_1_ have been proposed, the variability observed between felid species in the percentage of G_0_/G_1_ cells, an increase in cells in sub‐G_0_/G_1_, or a reduction in cell viability (Młodawska et al. 2022), has generated interest among different groups to better understand the reasons for these responses. This review describes the primary findings, challenges, and perspectives of G_0_/G_1_ synchronization methodologies used in SCNT and iPSC technologies in wild felids.

## 2 Challenges of G_0_/G_1_ Cell Synchronization

Obtaining a fibroblast line, which involves cell isolation and storage, is the first step toward synchronizing the cell cycle in G_0_/G_1_ (Bolton et al. 2022). In this step, three parameters are essential: (i) choosing the least‐invasive region of the animal for sampling, (ii) transport time, and (iii) aseptic conditions. These are critical challenges in recovering viable cells from animals with natural resources (Sano et al. 2022).

Aseptic conditions have been a challenge when establishing cell lines for species, including the jaguar (*Panthera onca*), but this can be addressed by better preparation of biopsy specimens under aseptic conditions and medium color control, in which the culture dish should be discarded if the medium changes to yellow (Mestre–Citrinovitz et al. 2016). Confirmation of the absence of contamination is important for cell applications, and this has been analyzed in several species, such as the Siberian tiger (*Panthera tigris altaica*) (Liu et al. 2010) and Bengal tiger (*Panthera tigris tigris*) (Guan et al. 2010). Moreover, transport and storage time influences the recovery of fishing cats (*Prionailurus viverrinus*) from natural resources when the tissue is stored for 1–3 days at 4°C (Sukparangsi et al. 2022).

Moreover, in vitro culture conditions can affect cell quality through the damage caused by oxidative stress and the number of passages (Place et al. 2017). In fishing cats, the removal of GlutaMAX reduces cell metabolism, with cells affected at the fifth passage (Sukparangsi et al. 2022) because of their lower density. In contrast, puma (*Puma concolor*) medium without supplementation promotes cell proliferation until the 10th passage (Lira et al. 2022). Variability in the cell culture media of other wild felids is shown in Table 1.

**Table 1:** Studies about the culture conditions and cell proliferation of fibroblasts derived from wild felids.

Species	Culture medium	Passage number	PDT (in h)	Growth curve	Authors
*Panthera tigris altaica*	DMEM supplemented with 10% FBS, and 1% non–essential amino acid	8th passage	17.2	Exponential phase until 144 h and stationary phase in 168 h	Song et al. (2007)
*Lynx pardinus*	DMEM supplemented with 1% non–essential amino acids, 0.1 mM 2–mercaptoethanol, 1 mM sodium pyruvate, 100 U/mL penicillin, and 0.1 mg/mL streptomycin	1st passage	48 at 240	Growth curve was not analyzed	León‐Quinto et al. (2011)
*Panthera leo persica*	DMEM supplemented with 20% FBS, and 2% antibiotics	5th passage	26.7	Exponential phase until 168 h and stationary phrase in 168 h	Yelisetti et al. (2016)
*Panthera tigris tigris*			27.2	Exponential until 144 h and stationary phrase after 192 h	
*Panthera pardus fusca*			34.7	The exponential phase until 144 h and stationary phrase after 168 h	
*Leopardus tigrinus*	DMEM supplemented with 10% FBS, with 2% penicillin G and streptomycin	3rd passage	24	Exponential phase after 120 h	Arantes et al. (2021)
*Leopardus colocolo*			24	Exponential phase after 192 h	
*Panthera onca*			24	Exponential phase until 70 h and stationary phase after 80 h	
*Panthera onca*	DMEM supplemented with 10% FBS, and 2% antibiotic/antimycotic solution	1st, 3rd, and 10th passage	22.8 at 26	The exponential phase until 72 h, stationary phase until 120 h and cell decrease after 144 h	Silva et al. (2021)
*Puma concolor*	DMEM supplemented with 10% FBS, and 2% antibiotic/antimycotic solution	1st, 3rd, and 10th passage	31	Exponential phase until 96 h, stationary phase until 144 h and decrease on 168 h	Lira et al. (2022)

Abbreviations: DMEM, Dulbecco's modified eagles medium; FBS, fetal bovine serum.

In this sense, specific characterization of the cell line may be the key to successful cycle synchronization, because knowledge of each species' doubling time and cell dynamics allows the appropriate use of cycle synchronization protocols. Arantes et al. (2021) described cell isolation from three wild felids: jaguar, northern tiger cat (*Leopardus tigrinus*), and pampas cat (*Leopardus colocolo*). The three species showed differences in their cellular ultrastructure and growth curves. Several other studies have reported variations in these characteristics among wild felids (Table 1).

## 3 Methodologies for G_0_/G_1_ Cell Synchronization

**Figure 1:**
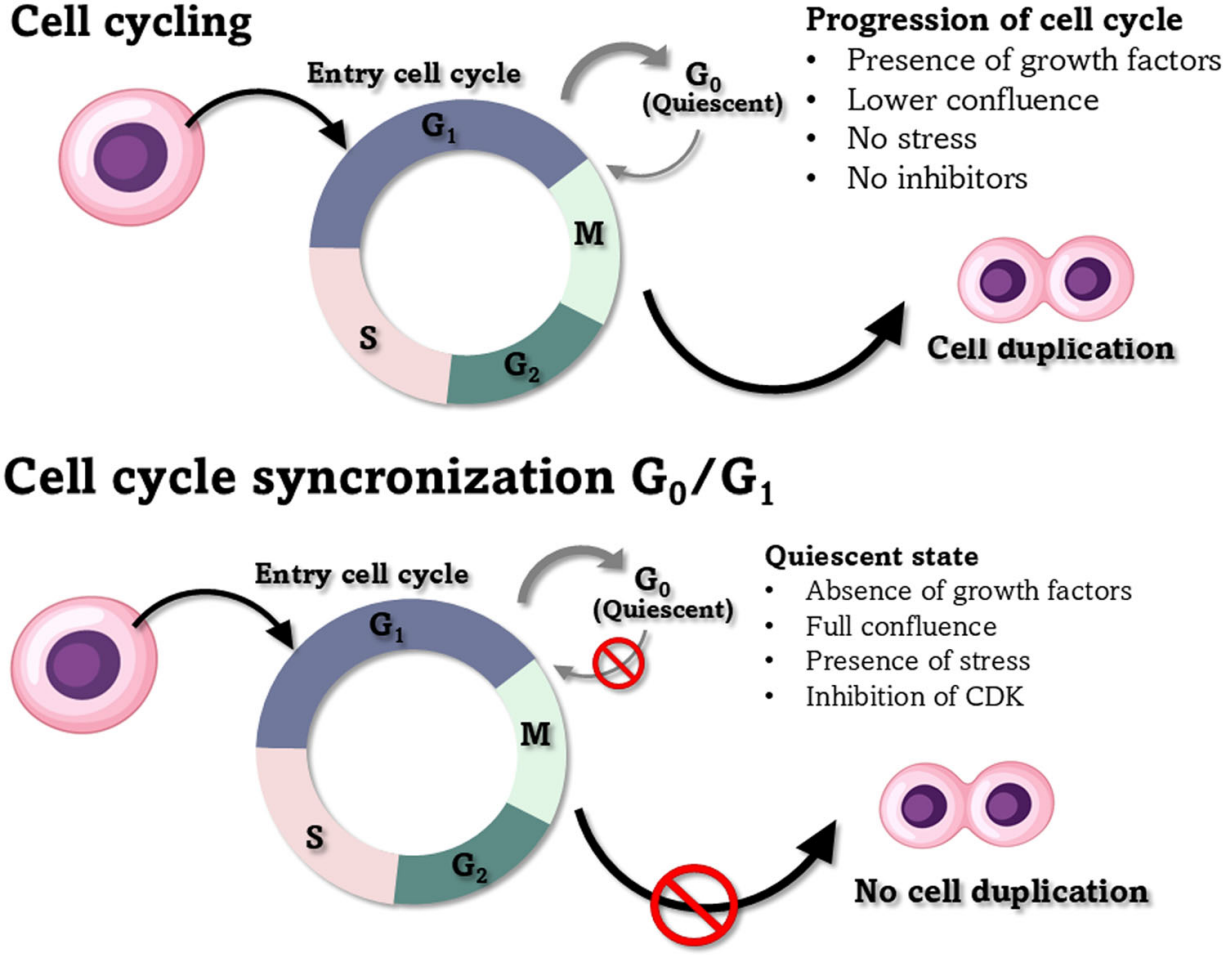
Representative scheme of the cell cycle and cells subjected to synchronization in G_0_/G_1_.

Cell cycle synchronization has been described as a method that can block proliferation under cell culture conditions or inhibit specific molecules (e.g., CDK), and this block should be reversible and nontoxic (Banfalvi 2017). Cells normally have checkpoints in the cell cycle; however, synchronized cells are unable to enter the cell cycle because of the absence of growth factors and the inhibition of CDK (Davis et al. 2001). An example of cell cycle synchronization conditions is shown in Figure 1.

Cells typically present an asynchronous cycle during in vitro culture, thus highlighting the importance of cell cycle synchronization methodologies for SCNT studies (Cooper 2003) and the uniformity of iPSC (Leal et al. 2024). These methods can be used in the presence of chemical agents that act through molecular inhibition or in the absence of chemical components through nutritional deprivation (SS) or CI (Eastman and Guo 2020).

In wild felids, roscovitine, which inhibits CDK, is most studied at concentrations from 7.5 to 30 µM, with incubation times from 12 to 24 h (Wittayarat et al. 2013; Rodrigues et al. 2023). Furthermore, Yelisetti et al. (2016) showed interesting results with the inhibition of histone deacetylase by sodium butyrate at concentrations of approximately 0.5 to 3.0 mM for 24 or 48 h. Hashem et al. (2007) highlighted the possibility of using a protease inhibitor (6‐dimethylaminopurine [6‐DMAP]), cycloheximide, cytochalasin B, and antioxidants (β‐mercaptoethanol, cysteine, and glutathione) for 4 h. With significant variability, it is essential to determine the ideal chemistry and incubation time for each species.

Without a chemical agent, nutrient deprivation inhibits the growth stimulus and promotes a quiescent state in G_0_/G_1_ (Davis et al. 2001). Several studies have shown effective results with SS by changing the concentration of fetal bovine serum (FBS) in the medium from 10% to 0.5% for 24–120 h (Yelisetti et al. 2016; Veraguas et al. 2017). The quiescent state can also be induced by stress caused by cell contact at 100% confluence and nutrient deprivation for 24 to 120 h (Wittayarat et al. 2013; Rodrigues et al. 2023). The combination of the two methodologies has shown effectiveness, with values of approximately 90% G_0_/G_1_ cells in *Puma yagouaroundi*, after incubation times of 24 to 120 h (Młodawska et al. 2022).

### 3.1 Chemical Inhibitors

Chemical inhibitors have been developed based on the molecule responsible for the regulation of the cell cycle, which is the most conserved molecule in the CDK group, where CDK1 plays an important role in cell cycle progression in combination with other CDKs (Krasinska et al. 2008). CDK inhibitors include roscovitine, which specifically inhibits the binding of CDK 1, 2, 5, and 7 to the ATP regions of molecules, with lower concentrations correlated with less cytotoxic effects (Wu et al. 2008).

The effects of roscovitine have been described in wild felids (Table 2), with efficiency observed only in the African wild cat (*Felis silvestris libica*) and Asian golden cat (*Pardofelis temminckii*). This has led to the development of cloned blastocysts of African wild cats (Gómez et al. 2003). In general, the incubation time was 12–24 h. Therefore, increasing concentration may be a viable strategy. However, Wittayarat et al. (2013) observed that 30 µM caused the most apoptotic cells in the Asian golden cat and leopard (*Panthera pardus*). In summary, new protocols should be developed based on the results observed in other wild felids with changes in the incubation time, between 12 and 24 h, and the concentration around 7.5, 15, and 30 µM.

**Table 2:** Studies using chemical agents in wild felid cells for G_0_/G_1_ cycle synchronization.

Chemical	Species	Better concentration	Time (in h)	Sub–G_0_/G_1_ cells (in %)	G_0_/G_1_ cells (in %)	Authors
Roscovitine	*Felis silvestris libica*	15 µM	24	—	~56	Gómez et al. (2003)
	*Pardofelis temminckii*	7.5 or 15 µM	24	~2	~93	Wittayarat et al. (2013)
	*Pardofelis marmorata*	7.5; 15 or 30 µM (No effect for species)		~1	~80	
	*Panthera pardus*	7.5; 15 or 30 µM (No effect for species)		~1	~80	
	*Puma concolor*	15 µM (No effect for species)	12 or 24	~2	~80	Rodrigues et al. (2023)
Sodium butyrate	*Panthera leo persica*	0.5 mM	48	~0.5	~76	Yelisetti et al. (2016)
	*Panthera tigris tigris*	0.5 mM	24	~0.3	~65	
	*Panthera pardus fusca*	0.5 mM	48	~0.5	~73	
ß–mercaptoetanol	*Panthera tigris altaica*	10 µM	4	—	~60	Hashem et al. (2007)
Cysteine		2 mM			~55	
Glutathione		2 mM			~73	
6‐DMAP		2 mM			~60	
Cycloheximide		7.5 µg/mL			~75	
Cytochalasin B		7.5 µg/mL			~61	

*Note:* –, Not analyzed in this study.

Alternative methods, such as sodium butyrate, a histone deacetylase (HDAC) inhibitor, have been tested in wild felids and have shown a significant increase in the percentage of synchronized cells to approximately 70% of cells in G_0_/G_1_ (Table 2). Yelisetti et al. (2016) showed no toxic effect of 1.0 mM sodium butyrate with 48 h of incubation for the Asiatic lion (*Phantera leo persia*), Indian tiger (*Phantera tigris tigris*), and Indian leopard (*Phantera pardus fusca*) in interspecies nuclear transfer (iSCNT), with approximately 5% blastocysts.

Hashem et al. (2007) tested the ability of antioxidants to increase oxidative stress and cell cycle interference, including a protein kinase inhibitor (6‐DMAP), a protein synthesis inhibitor (cycloheximide), and a cell division inhibitor (cytochalasin B). Alternative chemical tests are interestingly used strategies that use different methodologies to block cell‐cycle synchronization.

They will help us understand the best methodology for improving cell synchronization. Therefore, the chemical mechanism is essential to avoid DNA damage, the proapoptotic gene *BAX*, decreased viability, and cell detachment, as shown by methods, such as CI and SS (Veraguas et al. 2017).

### 3.2 Serum Starvation and Contact Inhibition

The SS method at 0.5% FBS for 120 h was the first used to clone sheep (Wilmut et al. 1997). This method has some benefits, such as no special reagent requirements; the low cost of the change, as it is only a change in culture medium conditions; and its effectiveness with several cell types (Jackman and O'Connor 2001). The SS and CI methods mediate cell cycle arrest by inducing stress. Both methods increase the concentration of p27, an inhibitor of CDK, and arrest the cell cycle (Davis et al. 2001; Pack et al. 2019).

The use of SS and CI has been effective for most wild felid species (Table 3), with more significant variance in the times from 1 (24 h) to 5 days (120 h). In particular, SS results in close to 100% of synchronized cells, and thus, it may be considered more effective than chemical methods, which have values closer to 70%–80% of G_0_/G_1_ cells. Hashem et al. (2007) compared the SS and CI of the chemicals tested (Table 2) and reported higher of G_0_/G_1_ for CI values when compared to some chemicals. Embryo production via SCNT, SS, and CI has been effective in African wild cats (~21%–28% of embryos, Gómez et al. 2003), Asiatic lions, Indian tigers, and Indian leopards (~3%–4% of embryos, Yelisetti et al. 2016).

**Table 3:** Effect of SS and CI on G_0_/G_1_ cells of wild felids.

Species	Method of synchronization	Time of treatment (in h)	Sub G_0_/G_1_ cells (in %)	G_0_/G_1_ cells (in %)	Authors
*Felis silvestris libica*	SS	120	—	~97	Gómez et al. (2003)
	CI	120	—	~88	
*Panthera tigris altaica*	SS	120	—	~66	Hashem et al. (2007)
	CI	120	—	~68	
*Pardofelis temminckii*	SS	24, 48, 72, or 120	~2, 5	~95	Wittayarat et al. (2013)
	CI	120	~3	~95	
*Pardofelis marmorata*	SS	48 or 72	~2	~95	
	CI	120 (No effect for species)	~2	~83	
*Panthera pardus*	SS	48, 72, or 120	~2	~95	
	CI	120	~1	~90	
*Panthera leo persica*	SS	24	~1,6	~85	Yelisetti et al. (2016)
	CI	24	~1.2	~91	
*Panthera tigris tigris*	SS	24	~1	~90	
	CI	24	~2	~92	
*Panthera pardus fusca*	SS	24	~1	~88	
	CI	24	~2	~90	
*Leopardus guigna*	SS	120	~2	~83	Veraguas et al. (2017)
	CI	24, 72, and 120 (not effective)	~2	~72	
*Puma yagouaroundi*	SS	120	—	~97	Młodawska et al. (2022)
	CI	72	—	~94	
*Felis manul*	SS	24	—	~95	
	CI	24	—	~93	
*Puma concolor*	SS	96	~3	~86	Rodrigues et al. (2023)
	CI	24	~6	~85	

*Note:* –, Not analyzed in this study.

Despite these promising results, alternative methods of cell synchronization are essential because the SS and CI methods can increase the number of apoptotic cells, and stress causes an increase in autophagy, senescence, and DNA fragmentation (Młodawska et al. 2022). Veraguas et al. (2017) described the effect of CI and SS on the relationship between *BAX* and *BCL2* genes, reporting an increase in the expression levels of these genes after 3 and 5 days of treatment. This was especially true for CI, which was considered to be ineffective because of its effect on viability.

### 3.3 Cell Synchronization as a Step for Somatic Cell Applications

**Figure 2:**
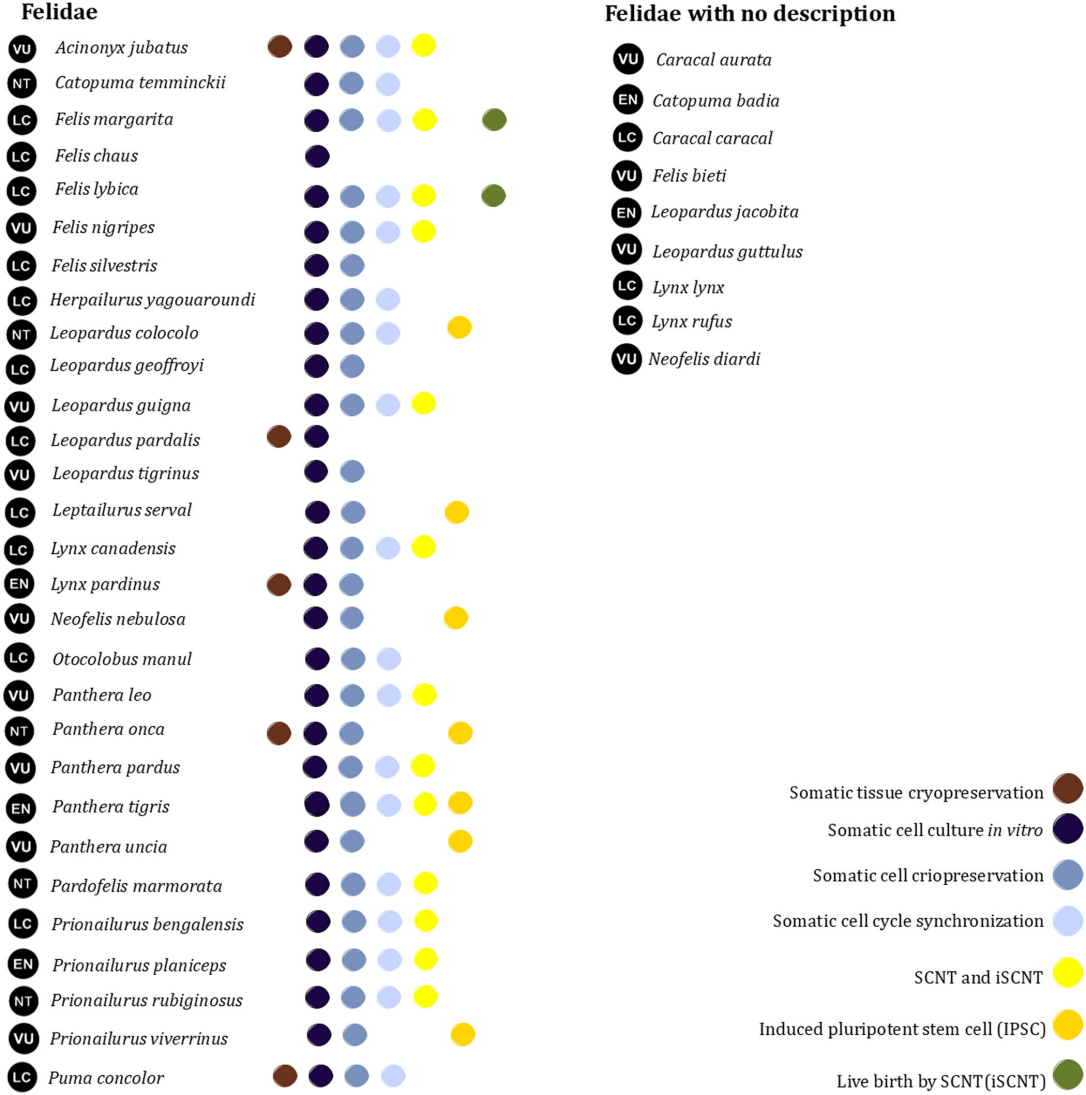
Studies of wild felids using somatic tissue and cells.

The applicability of SCNTs and iPSCs for somatic cells has been widely described. Several steps are performed during these procedures (Figure 2). The first steps for SCNT and iPSCs have been studied using cell culture and cryopreservation in 27 felid species. The next step is cell cycle synchronization, which has been performed for 18 species. Similarly, SCNT has been studied in only 14 species and only two species have shown birth after SCNT. These results are because of the complexity and multiple factors involved in iPSC and SCNT studies. Postmortem somatic tissue cryopreservation can also be used for cell recovery and applications in SCNT (Moulavi et al. 2017). However, this step has been established for only five species. Additionally, none of the studies on the use of iPSCs in wild felids have established a correlation between cell cycle synchronization and improvements in the results of iPSC use.

### 3.4 Correlation Between SCNT Efficiency and Cell Cycle Synchronization

The first step in the development of cloned embryos is to determine the quality of the nuclear donor and recipient oocytes. During this process, the nuclear donor should stay synchronized with the recipient for the correct nuclear reprogramming of cells (Swegen et al. 2023). The quiescent state promoted during the G_0_/G_1_ phase makes it possible to correct DNA replication and the accessibility of chromatin in the oocytes, which, with high quantities of maturation‐promoting factors when activated, promotes DNA exposition at the replication factors of oocytes and reprogramming (Gouveia et al. 2020).

Table 3 describes iSCNT studies involving SS and CI, with successful results indicating the possibility of blastocyst development and animal birth (Gómez et al. 2004). When comparing chemical agents with SS or CI for SCNT, only sodium butyrate demonstrated higher effectiveness than CI in lion blastocyst production (Yelisetti et al. 2016). However, the effect of roscovitine on wild felid blastocysts has not been well documented, and roscovitine does not yield better results than CI or SS.

In summary, even in Siberian tigers, where all methods have resulted in less than 70% of synchronized cells, SS and CI were the methods of choice for blastocyst production (Hashem et al. 2007); however, they resulted in the development of only one or two blastocysts. Reviewing the findings of previous studies to determine the best methodology for cell synchronization is essential because SS and CI are not effective for some species and chemical agents are not as effective as SS and CI for some species.

### 3.5 Can Cell Cycle Synchronization Improve the iPSC Generation on Wild Felids?

iPSCs can be reprogrammed using transcription factors such as OCT4, SOX2, KLF4, C–MYC, and NANOG (Verma et al. 2012, 2013). Variability among wild species in the effectiveness of different viral vectors, specific factor combinations, and media for iPSC proliferation in vitro has been described (Kumar et al. 2021). iPSCs have been developed for wild felids with the aim of applying these cells to germ cell development, genetic material restoration, and regenerative medicine (Liu et al. 2020).

Studies of iPSCs in wild felids have consisted of testing transfection methods with combinations of factors and assessing cell culture media and different coating surfaces. However, these studies have failed to produce fishing cat iPSCs (Sukparangsi et al. 2022). Verma et al. (2012, 2013) obtained iPSCs for the snow leopard (*Panthera uncia*), Bengal tiger (*Panthera tigris*), serval (*Leptailurus serval*), and jaguar and found no silenced factors after 14 passages. Moreover, other studies have focused on the effects of various factors (Verma et al. 2012, 2013), media, and vectors (Sukparangsi et al. 2022). To date, no studies have been conducted on the effects of cell synchronization on iPSC generation.

Moreover, the quiescent state of synchronized cells can improve cell reprogramming by inhibiting CDK, because the addition of CDK decreases the efficiency of cell reprogramming (Hindley and Philpott 2013). Therefore, studies on cell cycle synchronization during iPSC generation for wild felids are necessary, especially in species for which the insertion of reprogramming factors, such as the fishing cat, is not effective.

## 4 Conclusions

Cell cycle synchronization varies among wild felids, with specific methods of choice for each species. Successful cell cycle synchronization requires a high‐quality cell line without chromosomal damage, bacterial contamination, or senescence. In general, SS and CI methods have been the most effective for a higher number of species and have been most frequently applied to SCNT compared to chemical methodologies. Moreover, variability in the type of chemical, type of inhibition, incubation time, and concentration requires further study to increase the efficiency of this method. Finally, cell cycle synchronization is necessary for SCNT; however, the correlation between this step and wild‐type iPSC production has not been widely studied. Thus, the establishment of cell cycle synchronization must be studied, and the chemical methods and correlations between this step and the capacity to improve iPSC production should be improved.

## Ethics Statement

The authors have nothing to report.

## Conflicts of Interest

The authors declare no conflicts of interest.

## Data Availability

The data that support the findings of this study are available from the corresponding author upon reasonable request.
